# Epidemiology Trend of Chronic Kidney Disease in a Semi-Urban Tertiary Hospital in Sub-Saharan Africa

**DOI:** 10.7759/cureus.36912

**Published:** 2023-03-30

**Authors:** Henry Ovwasa, Henry O Aiwuyo, Ogochukwu C Okoye A., Evelyn Unuigbe, Nilum Rajora

**Affiliations:** 1 Family Physician, Milk River Health Center, Alberta, CAN; 2 Internal Medicine, Brookdale University Hospital Medical Center, Brooklyn, USA; 3 Nephrology, Delta State University Teaching Hospital (DELSUTH), Oghara, NGA; 4 Nephrology, University of Benin Teaching Hospital, Benin City, NGA; 5 Internal Medicine, University of Southwestern Medical Center, Dallas, USA

**Keywords:** change in trend, tertiary hospital, semi-urban, chronic kidney disease, epidemiology

## Abstract

Background

The global burden of chronic kidney disease (CKD) has been on an alarming increase in the last two decades. The morbidity and mortality associated with CKD are even worse in Nigeria, like other developing countries, due to multiple socioeconomic and demographic factors in the country. CKD contributes to the increasing need for hospital admission. Hypertension and chronic glomerulonephritis have been the leading causes of CKD in Nigeria. However, diabetic nephropathy has recently gained more significance as a cause of CKD in developing countries.

Aim and methods

This study aimed to describe the current trend in the burden and population characteristics of CKD in Southern Nigeria. This is a cross-sectional, hospital-based study. The study recruited adult patients with prehemodialysis CKD seen in renal clinics over a two-year period (November 2014 to October 2016). Data were obtained using a questionnaire and from the clinic register. All participants were clinically assessed, including history, anthropometric measurements, and urinary albumin-creatinine ratio.

Results

A total of 1,549 patients were seen at the Medical Outpatient Clinic over the study period. CKD accounted for 9.7% of medical outpatient clinic attendance. The mean age of participants was 49±13 years. The leading causes of CKD were diabetes mellitus (32%), chronic glomerulonephritis (30%), and hypertension (22%). Among the participants, CKD stages 3, 4, and 5 were prevalent in 26.7%, 43.3%, and 14.7%, respectively.

Conclusion and recommendation

CKD is very prevalent among medical clinic patients. Diabetic nephropathy seems to be a more significant cause of CKD than was previously reported. Late presentation of patients to nephrologists remains an obstacle to improving CKD outcome in Nigeria. There is need for more intensive preventive measures and early intervention.

## Introduction

The global burden of chronic kidney disease (CKD) has been on an alarming increase in the last two decades [1]. The morbidity and mortality associated with CKD in Nigeria and other developing countries in Sub-Saharan Africa (SSA) like Chad, Niger, Mali, and Mauritania are even worse due to the socioeconomic and demographic peculiarities of CKD patients in these countries [2,3]. The increasing burden of CKD in SSA is largely due to the rising prevalence of non-communicable diseases, such as hypertension, diabetes, and obesity, and the HIV/AIDS complications [4]. Akinsola et al. reported far back in 1989 that CKD accounts for 8-10% of hospital admission [5]. This, of course, does not represent the true magnitude of the problem as the vast majority of CKD patients cannot afford and access renal care in Nigeria due to a combination of illiteracy, poverty, inversely skewed distribution of renal services, and lack of social security system in the country [6,7].

A South-South Nigerian community study among rural adults reported a prevalence of CKD of 27.2% [8]. Thus, CKD is both grossly under-diagnosed and under-reported in hospital-based studies. Several Nigerian studies have shown that hypertension, chronic glomerulonephritis, and diabetes mellitus are the leading causes of CKD [9,10]. However, the increasing significance of diabetes mellitus as an etiology of CKD has been documented in studies conducted in developing countries [11]. The mean age of CKD patients in Nigeria, as in other developing countries, is between the third and fifth decades of life [12,13]. This is in contrast to findings in the western countries in which more than 50% of the CKD population are aged 65 years and above [14,15].

Consequently, the economic burden of the disease is worse in SSA due to a higher indirect cost in the form of man hour loss at workplace. Several studies have reported a male predominance in the CKD population in Nigeria [16,17]. However, Nwogu et al. reported a female predominance of 3:1 in a study of renal admission patterns in a South-East Nigerian tertiary hospital [18]. Similarly, in an earlier South-East Nigerian community-based study, the mean age of CKD patients was significantly lower in males than females [19]. Male gender, apart from being a risk factor for CKD, is also associated with a higher prevalence of other independent risk factors of CKD, such as hypertension, diabetes, and smoking [20].

The cost of renal replacement therapy in Nigeria is highly prohibitive, leading to a more than 70% drop-out rate within one month of hemodialysis [21]. The catastrophic health expenditure in developing countries in which majority of health financing is from patients paying out of pocket leaves most homes impoverished [22]. Preventive measures are therefore the most cost-effective and realistic options available to resource-poor countries. Apart from adequate treatment of hypertension, diabetes, and lifestyle modification, early detection and treatment of microalbuminuria are viable portals of intervention to prevent and halt the progression of CKD. Persistent proteinuria is both a marker of glomerular damage and a predictor of progression of CKD [23,24]. Hence, this study is important as it aims to mitigate the burden of CKD in the region by creating a purview for intervention.

## Materials and methods

The study was conducted at the renal unit of the Department of Medicine, Delta State University Teaching Hospital (DELSUTH), Oghara, South-South Nigeria, from November 2014 to October 2016 (spanning a period of two years). DELSUTH is a state-owned teaching hospital established by the Delta State Government in 2010. The health facility has 230 beds and it serves as the major tertiary health institution in the state and also a referral center for a large catchment area in the Southern part of Nigeria, including Edo, Bayelsa, Ondo, and Anambra states.

This is a hospital-based descriptive cross-sectional study. Ethical clearance was obtained from the Ethics Committee of DELSUTH. Consenting CKD patients attending the two renal clinics were recruited consecutively. However, patients in KDIGO (Kidney Disease Improving Global Outcomes) CKD stage 1, those with acute-on-chronic kidney disease, and those already on dialysis were excluded to avoid classification bias. Estimated glomerular filtration rate was calculated using the Modification of Diet in Renal Disease (MDRD) equation. A structured interviewer-administered questionnaire (close-ended) was used to obtain information on demographic data and the health status of participants. All participants were subjected to a thorough clinical assessment with emphasis on past medical history, social history, drug history, anthropometric measurements, blood pressure measurement, fundoscopy, urinalysis, fasting blood glucose, serum creatinine, urinary albumin-creatinine ratio (UACR), hepatitis B virus, hepatitis C virus, HIV screening, and renal ultrasonography.

CKD was defined and classified according to the 2012 KDIGO guidelines [25]. Diabetic nephropathy was defined by the history of diabetes mellitus, presence of diabetic retinopathy on fundoscopy, proteinuria > 300 mg/g, and normal-sized or enlarged kidneys on ultrasonography [6]. Hypertensive nephrosclerosis was defined by a long history of hypertension in a patient older than 45 years, signs of long-standing hypertension, minimal proteinuria/hematuria, and bilateral shrunken kidneys [6]. Chronic glomerulonephritis was defined by history suggestive of renal impairment with the presence of signs of long-standing hypertension, presence of proteinuria, hematuria, and bilateral shrunken kidneys on renal ultrasonography [26]. HIV-associated nephropathy was defined as a confirmed HIV-positive patient, with moderate-to-severe proteinuria, normal or low blood pressure, minimal edema, and normal-sized or enlarged kidneys on renal ultrasonography [4]. Diabetes mellitus was defined as a self-report of diagnosis by a doctor or other health personnel and/or history of use of anti-diabetic agents, elevated fasting blood glucose greater than or equal to 126 mg/dL, or random blood glucose greater than or equal to 200 mg/dL with symptoms of hyperglycemia [27]. Hypertension was defined as systolic blood pressure equal to or greater than 140 mmHg and/or diastolic blood pressure equal to or greater than 90 mmHg [28]. Obesity was defined as BMI equal to or greater than 30 kg/m^2^ [29].

Statistical analysis

The Statistical Package for Social Sciences (SPSS) computing program version 22(IBM Corp., Armonk, NY) was used for data management and analysis. Frequency of parameters is presented in tables, graphs, and pie charts. All continuous variables are presented as mean± SD, whereas categorical variables are presented as percentages and proportions. Mean of continuous variables was compared using Student's t-test, whereas categorical variables were compared using the chi-square test.

## Results

Prevalence of CKD in the medical outpatient clinic

During the study period, 1,549 patients attended the Medical Outpatient Clinic. Predialysis CKD patients constituted 9.7% of the total number of medical outpatient clinic patients. This is represented in Table 1.

**Table 1:** Prevalence of chronic kidney disease in the medical outpatient clinic

	Male, n (%)	Female, n (%)	Total, n (%)
Medical outpatients	844 (54.5)	705 (45.5)	1549 (100)
Renal outpatients	92 (61.3)	58 (38.7)	150 (100)

Sociodemographic characteristics and health status of participants

The mean age of participants was 49±13 years. The mean age of males was 47±12 years, which was significantly lower than that of females (51±15 years; t=2.58, p=0.001). Of the patients, 89% were less than 65 years of age, and 46.7% were in the age group of 45-64 years. There were 92 males and 58 females, making a male-to-female ratio of 3:2. Of the participants, 45%, 12%, 20%, and 23% were traders, farmers, civil servants, and others (artisans, lawyers, engineers, students, and unemployed), respectively. Majority (60%) of respondents earned between 10,000 and 100,000 nairas (US$28- US$280) per month. Only 25 (16.7%) earned more than 100,000 nairas (US$280) per month, and 32 (23.3%) were either unemployed or earned less than 10,000 nairas (US$28) per month. This is shown in Table 2.

**Table 2:** Sociodemographic characteristics of participants

		Frequency (n)	Percentage (%)
Age	18-44	63	42.0
	45-64	70	46.7
	≥65	17	11.3
Sex	Male	92	61.3
	Female	58	38.7
Ethnicity	Urhobos	79	52.7
	Ijaws	17	11.3
	Itsekiris	12	8.0
	Non-Deltans	42	28.0
Marital status	Single	30	20.0
	Married	103	68.7
	Divorced	11	7.3
	Widowed	6	4.0
Occupation	Trader	62	41.3
	Class 1	14	9.3
	Class 2	38	25.0
	Class 3	32	21.4
	Class 4	4	2.7
Income	<10,000 nairas	30	20.0
	10,000-100,000 nairas	100	66.7
	>100,000 nairas	20	13.3

CKD risk factors among participants

Of the participants, 64 (42.7%) and 46 (30.7%) had a history of hypertension and diabetes, respectively. Based on urinary albumin excretion (UAE) rates, only 20 (13.3%) were normoalbuminuric, whereas 41.3% and 45.3% of participants had microalbuminuria and overt albuminuria, respectively. Of the study group, 107 (71%) had no history of smoking, 31 (21%) had a previous history of smoking, and 12 (8%) had a current history of smoking. Also, 32 (21.3%) had a significant history of nephrotoxic agent use, 12 (8%) had a family history of kidney disease, and 28.7% had BMI equal to or greater than 30 kg/m^2^ (Table 3).

**Table 3:** Risk factors of chronic kidney disease among participants

	Frequency (n)	Percentage (%)
History of hypertension	64	42.7
History of diabetes	46	30.7
Smoking		
Current smokers	12	8.0
Previous smokers	31	20.7
Non-smokers	107	71.3
Obesity	43	28.7
Use of nephrotoxic agents	32	21.3
Family history of renal disease	12	8.0
Albuminuria		
Normal	20	13.3
Microalbuminuria	62	41.3
Overt proteinuria	68	45.3

Etiology and stages of CKD among participants

Diabetic nephropathy, chronic glomerulonephritis, and hypertensive nephrosclerosis were the leading causes of CKD in this population, accounting for 32%, 30%, and 22.6%, respectively. A total of 13 (8.6%) cases could not be attributed to a particular etiology, and 87 (58%) of the participants presented in either CKD stage 4 or 5. Only 23 (15.3%) cases presented in stage 2, as shown in Table 4.

**Table 4:** Etiology and stages of CKD among participants (N=150) CKD, chronic kidney disease; HIVAN, HIV-associated nephropathy

Etiology of CKD	Number of participants, n (%)
Diabetic nephropathy	48 (32%)
Chronic glomerulonephritis	45 (30%)
Hypertensive nephrosclerosis	34 (22.7%)
HIVAN	6 (4%)
Obstructive nephropathy	4 (2.6%)
Unknown etiology	13 (8.7%)

## Discussion

This study showed that CKD accounts for a high proportion of clinic patients seen in our medical outpatient clinic. The 9.7% prevalence of CKD among medical outpatient clinic patients is comparable with an earlier South-West Nigerian study, which reported CKD to account for 8-10% of medical admission [5]. The prevalence of CKD in this study is not a true reflection of the burden of the disease because most sufferers of CKD either do not present to the hospital or present at a very late stage of the disease. In a community-based study by Okoye et al., the prevalence of CKD was 27.2% [8]. Thus, CKD burden seen in the hospital setting constitutes just a tip of the iceberg of the actual enormity of the condition. The mean age of participants of 49±13 years is consistent with other studies that reported mean age of CKD patients between the third and fifth decades of life [12,13]. This, however, contrasts with reports from the western countries in which more than 50% of the CKD population are aged 65 years and above [14,15]. Findings in this study were consistent with the general knowledge that CKD is more common in young and middle-aged population in most developing countries. The implication of this observation is that the economic burden of the disease is worse in SSA, including Nigeria, due to a higher indirect cost in form of man hour loss at workplace.

The observation of the mean age of male CKD patients being lower than that of their female counterparts is similar to an earlier report by Ulasi and Ijoma in a South-East Nigerian study [19]. The reason for this observation is not clear-cut but may be related to the higher prevalence of risk factors of CKD in males as well as male gender being an independent risk factor itself [20]. The male-to-female ratio in this study was 3:2. This finding contrasts with earlier report of female-to-male ratio of 3:1 by Nwogu et al. in a South-East Nigerian teaching hospital study of patterns of renal admission [18]. Male dominance among CKD patients could be associated with the high financial burden and negative socioeconomic status of affected families and country by extension, especially in disadvantaged populations where males are more likely to be breadwinners of their homes.

The predominant occupation of participants was trading. While this could be a coincidence, the sedentary lifestyle associated with trading may contribute to CKD risk factors such as hypertension, diabetes mellitus, and obesity. Most participants in this study earned between 10,000 and 100,000 nairas ($28 and $280) per month. The catastrophic health spending in the developing countries wherein individuals mostly pay for Medicare out of pocket will undoubtedly further impoverish this CKD population with incomes already below the poverty line [22]. It is therefore not surprising that more than 70% of CKD patients on hemodialysis drop out within one month [21]. This underscores the need for more emphasis on preventive strategies and early detection of CKD rather than renal replacement therapy, which, in the developing countries, has been more of an exercise in futility.

The prevalence of hypertension in this study of 42.7% falls within the overall prevalence in Nigeria of 8.0% to 46.4% [6]. Hypertension is both a common cause and complication of CKD. The prevalence of hyperglycemia in this study of 30.7% is much higher than the 7.9% reported in an earlier study in South-South Nigeria but lower than that from the United States Renal Data System (USRDS) data, which reported diabetes mellitus in 54% cases of incident CKD [8,15]. The disparity in these observations could be a result of our study being hospital-based compared with the earlier community-based study. Diabetes is the leading cause of CKD worldwide and one of the leading causes of SSA [11]. The prevalence of overt albuminuria and microalbuminuria observed was 45.3% and 41.3%, respectively, in this study and was consistent with earlier study by Odili but significantly lower than the prevalence reported by Odum and Udi in a South-South Nigerian study [23,24]. The high prevalence of microalbuminuria underscores the limitation of the use of dipstick in evaluating UAE. Persistent proteinuria is both a marker of glomerular damage and a predictor of progression of CKD [23].

The leading causes of CKD in this study were diabetes nephropathy, chronic glomerulonephritis, and hypertensive nephrosclerosis, accounting for 32%, 30%, and 22.6%, respectively. Of those with diabetic nephropathy, 11% had no prior history of diabetes before presentation. This observation is both surprising and concerning as most earlier reports in Nigeria have observed much less significance of diabetes as an etiology of CKD [10,12]. However, while diabetes has remained the leading cause of CKD in the western countries, there has been an increasing prevalence of diabetes as an etiology of CKD in developing countries [11]. This epidemiologic transition is most likely due to a rising overall prevalence of diabetes mellitus, lifestyle westernization, and improvement in the survival of diabetics in developing countries such as Nigeria. Early detection of diabetic nephropathy and aggressive antiproteinuric measures will substantially reduce progression and improve overall outcome of diabetic renal disease. More than half of the participants presented in CKD stage 4 or 5. This is consistent with an earlier report by Arodiwe et al. in a South-East Nigerian study, in which majority of the patients studied presented in CKD stages 4 and 5 [9]. The implication of this observation is that CKD patients present late to the nephrologists in most developing countries, wherein complications of CKD are already advanced and largely irreversible.

This study, despite its strength of using the highly sensitive UACR in detecting UAE, had limitations, which included a one-time UACR, which did not differentiate between reversible and persistent proteinuria as well as being a single-center study, which will not allow generalization of findings. However, the findings in this study will stimulate future prospective studies on the subject.

## Conclusions

CKD is a major cause of medical outpatient consultation. Diabetic nephropathy seems a more significant etiology of CKD than was previously reported. Late presentation of CKD patients to nephrologists continues to be a norm in the developing countries. This calls for a concerted effort to raise awareness and implement programs for early screening and diagnosis of diabetes and CKD, especially in the high-risk populations.
